# Association between early childhood caries and poverty in low and middle income countries

**DOI:** 10.1186/s12903-019-0997-9

**Published:** 2020-01-06

**Authors:** Morenike Oluwatoyin Folayan, Maha El Tantawi, Nourhan M. Aly, Ola B. Al-Batayneh, Robert J. Schroth, Jorge L. Castillo, Jorma I. Virtanen, Balgis O. Gaffar, Rosa Amalia, Arthur Kemoli, Ana Vulkovic, Carlos A. Feldens, the ECCAG

**Affiliations:** 1 Faculty of Dentistry, Obafemi Awolowo University, Ile-Ife, Nigeria; 2 Faculty of Dentistry, Alexandria University, Alexandria, Egypt; 3 Department of Preventive Dentistry, Jordan University of Science and Technology, PO Box 3030, Irbid, 22110 Jordan; 4 Department of Preventive Dental Science, College of Dentistry, and Departments of Paediatrics and Child Health and Community Health Sciences, Max Rady College of Medicine, Rady Faculty of Health Sciences, University of Manitoba, Winnipeg, Canada; 5 Facultad de Estomatología, Universidad Peruana Cayetano Heredia, Lima, Peru; 6 Department of Clinical Dentistry, University of Bergen, Bergen, Norway; 7 Department of Preventive Dental Sciences, College of Dentistry, Imam Abdulrahman bin Faisal University, Dammam, Saudi Arabia; 8 Department of Preventive and Community Dentistry, Faculty of Dentistry, Universitas Gadjah Mada Yogyakarta, Yogyakarta, Indonesia; 9 Department of Paediatric Dentistry and Orthodontics, University of Nairobi, Nairobi, Kenya; 10 Department of Pediatric and Preventive Dentistry, School of Dental Medicine, University of Belgrade, Belgrade, Serbia; 11 Department of Pediatric Dentistry, Universidade Luterana do Brasil, Canoas, Brazil

**Keywords:** Early childhood caries, Dimensions of poverty, Monetary poverty, Water, hygiene, sanitation, Low-income countries, Middle-income countries

## Abstract

**Background:**

The aim of this study was to assess the relationship between early childhood caries (ECC) in 3–5-year-old children, seven indicators of poverty and the indicator of monetary poverty in low- and middle-income countries (LICs, MICs).

**Methods:**

This ecologic study utilized 2007 to 2017 country-level data for LICs and MICs. Explanatory variables were seven indicators of poverty namely food, water, sanitation, health, shelter, access to information, education; and monetary poverty. The outcome variable was the percentage of 3–5-year-old children with ECC. A series of univariate general linear regression models were used to assess the relationship between the percentage of 3–5 year-old children with ECC and each of the seven indicators of poverty, and monetary poverty. This was followed by multivariable regression models to determined the combined effect of the seven indicators of poverty, as well as the combined effect of the seven indicators of poverty and monetary poverty. Adjusted R^2^ measured models’ ability to explain the variation among LICs and MICs in the percentage of 3–5-year-old children with ECC.

**Results:**

Significantly more people had food, sanitation, shelter, access to information, education and monetary poverty in LICs than in MICs. There was no difference in the prevalence of ECC in 3–5-year-old children between LICs and MICs. The combination of the seven indicators of poverty explained 15% of the variation in the percentage of 3–5-year-old children with ECC compared to 1% explained by monetary poverty. When the seven indicators of poverty and the indicator for monetary poverty were combined, the amount of variation explained by them was 10%. Only two of the poverty indicators had a direct relationship with the percentage of children with ECC; there was a higher percentage of ECC in countries with higher percentage of population living in slums (B = 0.35) and in those countries with higher percentage of the population living below poverty lines (B = 0.19). The other indicators had an inverse relationship.

**Conclusion:**

The use of multiple indicators to measures of poverty explained greater amount of variation in the percentage of 3–5-year-olds with ECC in LICs and MICs than using only the indicator for monetary poverty.

## Background

Early childhood caries (ECC) is a disease with high prevalence in many countries around the world. High sugar consumption is the primary risk factor [1]. There are multiple predisposing factors for ECC including those that increase the risk for high consumption of sugar, and those that increase tooth susceptibility to caries [2–5]. These factors have largely been studied at the individual level. Among the predisposing factors studied are parental socioeconomic, educational, income and employment status, as well as occupation [6–12]. These measures have often been used as proxy measures of poverty at the individual level [13–17]. There are a few contextual measures of poverty some of which have explored how the residential location affects the risk for ECC. Most of these studies had identified that more children living in poorer communities and in low-income household have ECC [18–24].

Recently, Baker et al. [25] and El Tantawi [26] identified a strong association between contextual variables and caries in adults and in preschool children respectively. Prior studies have demonstrated that access to and consumption of sugar is higher in low-resourced settings due to high sugar containing meals being a cheaper readily accessible alternative diet [27]. The prevalence of caries and the proportion of children with untreated caries being higher in poorer communities and lower in high-income countries suggest a possible relationship between ECC and poverty [6]. This relationship has not been a consistent finding in all studies. For example in Brazil, the Human Development Index, average household income and number of public primary healthcare units were not associated with the proportion of preschool children with untreated decayed teeth in the poorer neighborhoods in southern Brazil. However, these indicators were associated with the proportion of children with filled teeth living in richer areas [28]. This inconsistency in the few studies on poverty may be due to it being defined as a single construct.

There are seven dimensions of childhood poverty namely: food, water, sanitation, health, shelter, education and information [29]. These are derived from the United Nation’s measure of absolute poverty defined as a condition characterized by severe deprivation of basic human needs [30]. The experience of poverty is the result of a combination of different factors [31], which are multi-dimensional and interrelated [32].

The most frequently studied dimension of poverty related to ECC is food, which is assessed as nutritional status and dietary intake. While large population-based studies have not found an association between body mass index and ECC [33, 34], longitudinal studies suggest that there was an association between malnutrition and ECC [35, 36]. A few other studies demonstrated the association between ECC and access to health information [15], shelter [37] and education [38]. Studies investigating the relationship between shelter and caries in homeless children reported a high prevalence of ECC [39] though the prevalence of ECC in urban slum and rural India did not differ from that observed in other regions in India [40]. Meanwhile, there are no identified studies on the relationship between portable water, sanitation and ECC. The limited information on the association between dimensions of poverty and ECC creates a gap in knowledge. This knowledge is needed to enhance structural interventions to prevent or reduce diseases like ECC.

The aim of this study was to assess the relationship between ECC in 3–5-year-old children and multiple dimensions of poverty in low-income countries (LICs) and middle-income countries (MICs). For this study, the seven dimensions of poverty studied were food, water, sanitation, health, shelter, education, access to information in contrast to monetary poverty. The hypothesis was that the prevalence of ECC is higher in countries with higher poverty levels.

## Methods

This ecologic study was based on country-level data for LICs and MICs covering the period from 2007 to 2017. The Gross National Income per capita for 2017, calculated with the World Bank Atlas method [41] was used to define the countries’ economic level. Thus, LICs were defined as those with gross domestic product (GDP) of $995 or less; MICs were those with GDP of $996–$12,055. High-income countries were excluded from this study as there were no data available for the dimensions of poverty variables studied.

**Fig. 1:**
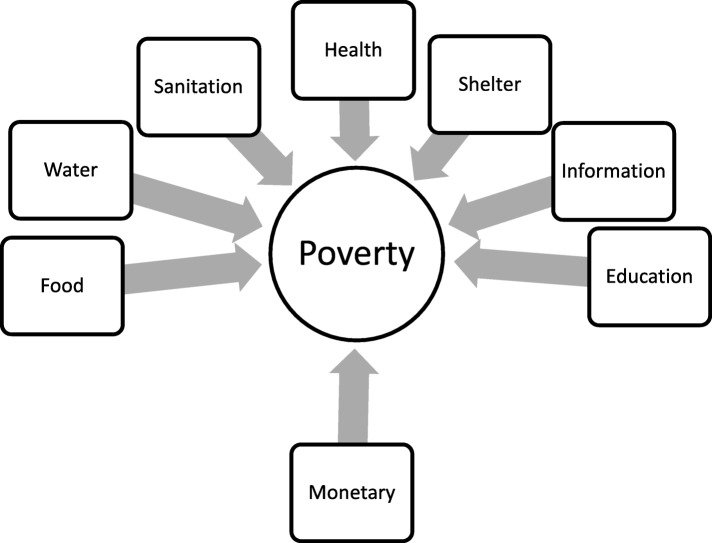
Diagrammatic presentation of the seven indicators of poverty and monetary poverty

Explanatory variables included indicators of the seven dimensions of poverty based on the Convention on the Rights of Children as developed by the University of Bristol and used in the UNICEF’s global study on child poverty [42]. The framework for the study is shown in Fig. 1. The various indicators and the definitions of the dimensions of poverty assessed in this study are presented in the supplemental file (Additional file 1: Appendix 1). Food related poverty was measured by the percentage of under 5-year-old children with moderate to severe underweight [43]; water poverty was measured by percentage of the population with surface water coverage [44], while sanitation poverty was measured by percentage of the population with open defecation [44]. Health-related poverty status was measured by the percentage of under 5-year-old children with diarrhea who received oral rehydration therapy (ORT) with continued feeding [45]; shelter poverty was measured by the percentage of urban population living in slums [46]; and information as the mobile cellular subscription per 100 persons [47]. Educational poverty was measured by the percentage of entrants to the last year in primary school relative to the total number of children in the same age as that for enrolling in that school grade [48]. Monetary poverty was measured by the percentage of the population below the national poverty line per country [49].

The outcome variable was the percentage of 3–5-year-old children with ECC. This data was collected in a previous study [26] through comprehensive search of the World Health Organization database and the existing literature without restriction by gender or language. The literature search used systematic methodology and was conducted in several databases reported in the primary study, using the definition of ECC adopted by the American Academy of Pediatric Dentistry: ECC was defined as one or more decayed, missing due to decay or filled primary tooth surfaces in children less than 72 months of age [50]. The percentage of children with ECC per country was calculated by adding the number of those affected by ECC in all relevant studies and dividing this by the total number of children examined and multiplying by hundred.

We calculated the number and percentage of LICs and MICs from which we were able to extract information on all the variables (Additional file 1: Appendix 2). The numerator for the percentage calculated was the number of the countries for which data for all the study variables were extracted. The denominator was the number of countries in each income category listed by the World Bank for 2017 [51].

We also calculated means and standard deviations for the indicators of poverty. The indicator for health (percentage of children receiving ORT and food after diarrhea), information (number of mobile subscriptions per 100 persons) and education (percentage of entrants to last grade of primary school) were reversed to reflect that the higher the prevalence the higher the poverty in line with the other measures of poverty used for this study. This was done by subtracting the percentage of those with ORT and feeding, those with mobile subscription and those who enrolled in the last primary grade from 100. Available values for the seven indicators of poverty and monetary poverty indicators were averaged for the period 2007 to 2017.

### Statistical analysis

The countries were categorized into two income levels - LICs and MICs, and the distribution of the study variables was compared between these two categories. The differences in significance were investigated using t-test or Mann Whitney U test after assessing the normality of distribution of the study variables.

A series of univariate general linear regression models were used to assess the relationship between the percentage of 3–5-year-old children with ECC and each of the explanatory variables one at a time (Model 1) followed by multivariable models. We assessed multi-collinearity and found no high correlations (*r* > 0.7) signifying no problem [52]. Based on this finding, we kept all variables to preserve the integrity of our conceptual framework. Model 2 included the seven indicators of poverty together. Model 3 assessed the relationship with monetary poverty. Model 4 included the seven indicators of poverty, and monetary poverty. We calculated regression coefficients (B) and 95% confidence intervals (CI) in addition to adjusted R^2^ as a measure of model ability to explain the variation among LICs and MICs in the percentage of 3–5-year-old children with ECC. Statistical analysis was performed using IBM SPSS for Windows version 22.0 (IBM Corp., Armonk, N.Y., USA). Significance level was set at <5%.

## Results

Table 1 highlights the number and percentage of countries in the two income levels included in the data analysis and the means and standard deviations for the indicators of poverty for each category. Overall, 11.8% of the included countries were LICs. LICs had a significantly higher mean percentage of <5-year-old children with moderate to severe underweight than MICs (mean = 5.90 and 3.20, *P* = 0.03). In addition, LICs had a significantly higher mean percentage of population that openly defecated (mean = 10.96 and 6.32, *P* = 0.03), lived in slums (mean = 53.19 and 34.65, *P* = 0.03), had no mobile cellular subscription per 100 persons (mean = 39.64 and 4.42, *P* = 0.005) and had primary non-completion rate (mean = 28.09 and 6.21, *P* = 0.009) than MICs. There were no significant differences between LICs and MICs in the percentage of the population with surface water coverage (*P* = 0.32) and percentage of <5-year-old children with diarrhea who did not receive ORT (*P* = 0.39). A significantly higher percentage of the population living below the national poverty line was found in LICs than in MICs (mean = 39.59 and 24.35, *P* = 0.009). There was no significant difference between LICs and MICs in the percentage of 3–5 year-old children with ECC (mean = 63.12 and 65.65, *P* = 0.75).

**Table 1:** Level of dimensions of poverty and monetary poverty in the low and middle-income countries included in the study

STUDY VARIABLES	LICs	MICs	*P* value
N (%)	6 (11.8)	45 (88.2)	–
Percentage of children <5 years with moderate to severe underweight: Mean (SD)	5.90 (2.93)	3.20 (3.38)	0.03
Percentage of the population with surface water coverage: Mean (SD)	5.52 (5.55)	3.01 (4.07)	0.32
Percentage of the population with open defecation: Mean (SD)	10.96 (7.02)	6.32 (10.41)	0.03
Percentage of children <5 years with diarrhea who did not receive ORT with continued feeding: Mean (SD)	52.71 (7.79)	47.21 (14.91)	0.39
Percentage of urban population living in slums: Mean (SD)	53.19 (13.02)	34.65 (18.34)	0.03
No mobile cellular subscription per 100 persons: Mean (SD)	39.64 (24.82)	4.42 (26.59)	0.005
Primary non-completion rate: Mean (SD)	28.09 (17.12)	6.21 (11.15)	0.009
Percentage of population below the national poverty line: Mean (SD)	39.59 (16.20)	24.35 (12.45)	0.009
Percentage of 3–5-year-old children with ECC: Mean (SD)	63.12 (20.33)	65.65 (17.85)	0.75

Table 2 shows the relationship between ECC and the seven indicators of poverty among 3–5-year-old children in the regression models. The combination of the seven indicators of poverty in Model 2 explained 15% of the variation in the percentage of 3–5 year-old children with ECC compared to 1% explained by the monetary poverty in Model 3. When all indicators were combined in Model 4, the amount of variation explained by them was lower (10%) than in Model 2 (15%).

**Table 2:** Relationship between ECC in 3–5-year-old children, the seven indicators of poverty and monetary poverty in the low and middle-income countries

	Model 1	Model 2	Model 3	Model 4
Percentage of children <5 years with moderate to severe underweight	−0.56 (−2.34, 1.21)	−0.46 (−4.29, 3.37)	–	− 0.85 (−5.20, 3.50)
Percentage of the population with surface water coverage	− 0.91 (− 2.12, 0.30)	− 0.56 (− 2.98, 1.85)	–	− 0.70 (−3.27, 1.88)
Percentage of the population with open defecation	−0.06 (− 0.58, 0.46)	−0.15 (−1.01, 0.70)	–	−0.09 (− 1.02, 0.83)
Percentage of children <5 with diarrhea who did not receive ORT with continued feeding	− 0.39 (− 0.80, 0.02)	−0.33 (− 0.91, 0.25)	–	− 0.26 (− 0.95, 0.43)
Percentage of urban population living in slums	−0.09 (− 0.43, 0.24)	0.29 (− 0.55, 1.14)	–	0.35 (− 0.56, 1.27)
No mobile cellular subscription per 100 persons	−0.12 (− 0.30, 0.06)	−0.41 (− 0.89, 0.07)	–	− 0.44 (− 0.95, 0.07)
Primary education non-completion rate	−0.30 (− 0.59, 0.002)	0.06 (− 0.65, 0.77)		−0.01 (− 0.80, 0.79)
Percentage of population below the national poverty line	−0.13 (− 0.52, 0.26)	–	− 0.13 (− 0.52, 0.26)	0.19 (− 0.68, 1.06)
Adjusted R^2^	–	0.15	0.01	0.10

Model 1: univariate- individual factors one at a time; Model 2: seven indicators of poverty (underweight, surface water, open defecation, no diarrhea treatment, living in slums, no cellular phone subscription and primary non-completion rate); Model 3: indicator of monetary poverty; Model 4: indicators in Models 2 and 3

R^2^ for each of the factor included in the univariate regression in Model 1 = 0.02, 0.03, 0.02, 0.07, 0.02, 0.02, 0.009 and 0.01

In Model 4, six of the seven indicators of poverty had an inverse relationship with the percentage of children with ECC (Table 2). There was lower percentage of children with ECC in countries with one unit higher percentage of children under 5-years of age with moderate to severe underweight (B = − 0.85), one unit higher percentage of population with surface water coverage (B = − 0.70), one unit higher percentage of population with open defecation facilities (B = − 0.09), one unit higher percentage of children with diarrhea who did not receive ORT (B = − 0.26), one more person in 100 with no mobile cellular subscription (B = − 0.44) and one unit higher percentage of children who did not complete primary school (B = − 0.01).

The remaining indicator - percentage of urban population living in slums - had a directly proportional relationship with the percentage of children with ECC: there wasa higher percentage of ECC in countries with one-unit higher percentage of urban population living in slums (B = 0.35). In addition, there was a higher percentage of ECC in countries with higher percentage of the population living below poverty lines (B = 0.19).

## Discussion

This study provides initial evidence on the relationship between ECC in 3–5-year-olds and various dimensions of poverty. We identified the association between country-level measure of poverty and ECC. In addition, we identified the relationship between measures of severe deprivation of basic human need, which are more likely to have serious adverse consequences for the health, development and well-being of children, and ECC. Our study highlights two important findings. First, not all the measures of poverty had the same relationship with ECC; while there was less ECC in countries with more poverty indicated by worse conditions of food, water, sanitation, health, information and education, ECC was more prevalent in countries with shelter and monetary poverty. Second, the combination of the seven indicators of poverty explained more variation than monetary poverty alone. This combination also explained more variation than combining all the seven indicators of poverty along with he tmonetary poverty indicator. The study hypothesis was therefore, only partially sustained.

One of the strengths of the study was its use of large datasets collated from measures derived from the global surveys such as the Demographic Health Survey and MIC, which provide high quality data [53]. We also did not use a summative index for poverty thereby reducing the risk for discounting item-level distinctions, and showing clearly that different measures of poverty do not have the same impact on ECC.

Despite the strengths of the study, the study findings need to be interpreted with caution because of the possible fallacies associated with ecological studies likes ours. The proxy measures of poverty were not all age specific. We also did not control for traditional risk factors for ECC like oral hygiene, sugar consumption, tooth brushing, use of fluoride and access to oral healthcare as these remain largely unknown due to lack of country-level data. In addition, because of data availability issues, our sample consisted of mostly MICs and few LICs. The differences in the distribution of poverty indicators between MICs and LICs may have affected our findings. We used data that generally targets non high-income countries (HICs) implying that our findings should not be generalized to HICs. In spite of the study limitations, the findings provide insights that can help in generating hypotheses for further studies on ECC and poverty.

In the present study, monetary poverty was associated with less ECC in univariate regression. However, when added to the other indicators of poverty, it was associated with more ECC. Our finding partly agrees with previous studies suggesting higher prevalence of ECC in resources-constrained settings when compared to high-income countries. Higher prevalence of ECC in resources-constrained settings results from poorer access to factors that may reduce diseases risk such as healthcare, professional advice, healthy dietary choices and preventive dental programme [54], and easier access to diets rich in free sugar because it is affordable [55].

We found that countries with higher percentage of its population living in urban slums had higher prevalence of ECC. There are more urban slums in transitioning economies; and transiting economies are associated with higher consumption of caries-promoting diets [56, 57]. Urban slums are also characterized by lack of basic infrastructure, dearth of socio-economic opportunities, extreme deprivation, and enduring marginalization [58]. The findings may reflect the poorer health status of children resident in slums compared to those in urban settlement [59]. The poorer health outcomes of children resident in slums may be related to the lower educational status of mothers in these settings [60]. Maternal education is a strong predictive variable for ECC, stronger than household income [61]. While household income represents the power to purchase material goods, maternal education represents the ability to provide care including regular dental care visits and not offering sugary foods to children, which are protective factors for ECC [49]. Although the pathways by which mother’s schooling affect caries is not fully understood, it is likely that health beliefs, locus of control and self-efficacy may at least partially explain these findings [62].

Also, children living in urban slums are more likely to be exposed to poor environmental factors, and have poor access to oral healthcare, and easy access to cheap sugary diets than health food thereby contributing to the high risk for ECC [63]. In addition, stress and poor residential stability associated with living in the slum could lead to less self-care including poor oral care for preschoolers [64]. There are few studies on ECC and urban slum residency [65] despite the growing literature on the health impact of the environment, urbanization and migration. Our study finding shows the need for further studies.

We found countries with sanitation, access to health, information and clean water problems had lower prevalence of ECC. We postulate that countries with sanitation, access to health, information and clean water challenges are likely to be poor indigenous communities still largely dependent on traditional agrarian diet with less consumption of industrialized and ultra-processed meals [66]. They are also more likely to be dealing with problems associated with infectious diseases rather than non-communicable disease (such as ECC) seen in countries with transitioning economies.

There are no studies determining the relationship between sanitation, and access to clean water and oral diseases in preschoolers. Studies are, therefore needed to understand the link between sanitation and caries. Water, sanitation and hygiene (WASH) programs reduce the risk of infectious diseases by creating environments that support good personal hygiene, and access to water and sanitation [67]. This improves the health of the population [68], and through that, improve country economy. Many countries with WASH programs are transitioning economies with changing dietary patterns that increase the risk of non-communicable diseases, including ECC [69]. If these countries do not concurrently address oral health issues, such as through universal health coverage programs [34], they may also experience the problem of ECC.

We also found that malnutrition resulting in moderate to severe underweight was not associated with higher prevalence of ECC unlike what some prior studies had indicated [69, 70]. A previous ecological study, which was more granular in its analysis on the relationship between malnutrition and prevalence of ECC in 3–5 year-olds, also showed no significant association between malnutrition and caries in the age group [71]. The debate on the relationship between malnutrition and the prevalence of ECC remains unresolved, and our findings only adds to the uncertainty about this relationship. A prospective cohort study may provide definite answers about the relationship.

The relationship between ECC and the dimensions of poverty observed in this study may be explained by the association between health and poverty in general. The improvement in the health status of countries is more dependent on how society organizes itself and uses all available resources rather than on wealth status per se. While the association between ECC and poverty indicators my not be of the same direction and strength for all dimensions, poverty is still an important social determinant for oral health and understanding how the dimensions inter-relate is important for planning and implementing cost-effective interventions [72, 73]. The present findings suggest that the impact of poverty on ECC goes beyond the availability of financial resources; it is also related to how financial resources are used to improve children’s life and comprehensively ensure their wellbeing. This may have implications for health education and policy setting where oral health services should be an integral part of planning for sustainable development. Designing longitudinal studies that builds on this hypothesis-generating ecological study may help oral health workers to better target their ECC preventive care for pre-school children identified to be at risk, using a defined set of poverty indicators.

## Conclusion

The study indicated that there is a complex relationship between multiple indicators of poverty and ECC. A combination of seven indicators of poverty explained greater amount of variation in the percentage of 3–5-year-olds with ECC in LICs and MICs than the indicator for monetary poverty did. Programs addressing poverty-related infrastructure problems like water access, improved sanitation and ORT after diarrhea may have negative implications for ECC control if not managed using a comprehensive approach that includes considerations for the children’s oral health. Most of the dimensions of poverty measured in this study are associated with problems of communicable diseases that are becoming less dominant in LICs and MICs giving way to a double burden of communicable and non-communicable diseases, with ECC being part of the latter. This may explain the negative association between most of the indicators of poverty and ECC prevalence observed in this study.

## Supplementary information

**Additional file 1.** Appendix 1. Indicators and definitions of multidimensional and monetary poverty used in the study. Appendix 2: List of countries included in the study.

## Data Availability

The datasets used and analysed for this study are publicly available.

## Abbreviations

ECC: Early Childhood Caries
GDP: Gross Domestic Product
LICs: Low-income Countries
MICs: Middle-income Countries
ORT: Oral Rehydration Therapy
WASH: Water, sanitation and hygiene
