# Supplementary material for: Association between early childhood caries and poverty in low and middle income countries
Source: BMC Oral Health. 2020 Jan 6;20:8. doi: 10.1186/s12903-019-0997-9 (PMC6945445; doi:10.1186/s12903-019-0997-9)
Supplement: Supplementary file 1 — Additional file 1. Appendix 1. Indicators and definitions of multidimensional and monetary poverty used in the study. Appendix 2: List of countries included in the study. [file 12903_2019_997_MOESM1_ESM.docx]

Supplemental file

Appendix 1: Indicators and definitions of multidimensional and monetary poverty used in the study

| Indicator | | Definition | Link |
| --- | --- | --- | --- |
| Food | Prevalence of Children Under 5 moderately to severely underweight  (chronic) | The percentage of children < 5 years old whose weights are <2 SD below the median weight for age groups in the international reference population. | <http://mdgs.un.org/unsd/mdg/Data.aspx> |
| Water | Surface water  % coverage | The percentage of the population drinking water directly from a river, dam, lake, pond, stream, canal or irrigation canal | <https://washdata.org/data/household#!/> |
| Sanitation | Open defecation  % coverage | The percentage of the population disposing of human feces in fields, forests, bushes, open bodies of water, beaches and other open spaces or with solid waste | <https://washdata.org/data/household#!/> |
| Health | Diarrhea treatment | The percentage of children < 5 year old not receiving oral rehydration therapy (ORT) with continued feeding after diarrhea in the two weeks prior to the survey | <https://data.worldbank.org/indicator/SH.STA.ORCF.ZS> |
| Shelter | Population living in slums (% of urban population) | The proportion of urban population living in slum households, which is a group of individuals living under the same roof lacking one or more of the following conditions: access to improved water, access to improved sanitation, sufficient living area, and durability of housing. | <https://data.worldbank.org/indicator/EN.POP.SLUM.UR.ZS> |
| Information | Mobile cellular subscriptions (per 100 people) | Mobile cellular telephone subscriptions are subscriptions to a public mobile telephone service providing access to the PSTN using cellular technology. The indicator includes the number of postpaid subscriptions, and active prepaid accounts. | <https://data.worldbank.org/indicator/IT.CEL.SETS.P2> |
| Education | Primary completion rate, total (% of relevant age group) | Primary completion rate is the number of new entrants in the last grade of primary education divided by the population at the entrance age for the last grade of primary education. | <https://data.worldbank.org/indicator/SE.PRM.CMPT.ZS?view=chart> |
| Monetary child poverty measure | Poverty headcount ratio at national poverty lines (% of population) | The percentage of the population living below the national poverty lines. National estimates are based on population-weighted subgroup estimates from household surveys. | <https://data.worldbank.org/indicator/SI.POV.NAHC> |

Appendix 2: List of countries included in the study

ID Country Income level

1 Albania MIC

2 Bosnia & Herzegovina MIC

3 Brazil MIC

4 Cambodia MIC

5 Colombia MIC

6 Congo, DRC LIC

7 China MIC

8 Ecuador MIC

9 Egypt MIC

10 El Salvador MIC

11 Gambia LIC

12 Georgia MIC

13 India MIC

14 Indonesia MIC

15 Iran MIC

16 Iraq MIC

17 Jamaica MIC

18 Kazakhstan MIC

19 Kenya MIC

20 Kyrgyzstan MIC

21 Laos MIC

22 Lebanon MIC

23 Libya MIC

24 Malaysia MIC

25 Mexico MIC

26 Mongolia MIC

27 Morocco MIC

28 Myanmar MIC

29 Namibia MIC

30 Nepal LIC

31 Nigeria MIC

32 Pakistan MIC

33 Paraguay MIC

34 Peru MIC

35 Philippines MIC

36 Romania MIC

37 Russia MIC

38 Senegal LIC

39 Serbia MIC

40 South Africa MIC

41 Sri Lanka MIC

42 North Sudan MIC

43 Syria MIC

44 Tanzania LIC

45 Thailand MIC

46 Turkey MIC

47 Uganda LIC

48 Ukraine MIC

49 Vanuatu MIC

50 Venezuela MIC

51 Vietnam MIC
